# Numerical Analysis and Design of an EMF Birdcage Wearable Device for the Treatment of Alzheimer’s Disease: A Feasibility Study

**DOI:** 10.4236/jbise.2022.158020

**Published:** 2022-08-17

**Authors:** Felipe P. Perez, David Michael Arvidson, Tyler Phoenix Taylor, Maryam Rahmani, Maher Rizkalla

**Affiliations:** 1 Department of Medicine, Division of General Internal Medicine and Geriatrics, Indiana University School of Medicine, Indianapolis, USA;; 2 Department of Electrical and Computer Engineering, Indiana University-Purdue University, Indianapolis, USA

**Keywords:** Alzheimer Disease, Birdcage, Treatment, HFSS, EMF, Temperature

## Abstract

In this study, we performed a numerical analysis of a novel EMF Birdcage wearable device for the treatment of Alzheimer’s disease (AD). We designed the new device to generate and radiate a frequency of 64 MHz and a specific absorption rate (SAR) of 0.6 W/kg to a simulated human brain tissue. We determined these parameters from our experimental studies on primary human brain cultures at the Indiana University School of Medicine (IUSM). We found that this frequency and SAR decreased the toxic A*β* levels in the cell cultures. The frequency of 64 MHZ has good skin depth penetration, which will easily pass through the various head layers, including hair, skin, fat, dura, the cerebrospinal (CSF), and grey matter, and reach deeply into the brain tissues. The SAR of 0.6 W/kg was achieved with lower power input and energy, decreasing the probability of thermal injury. Therefore, these parameters enhance the safety of these potential treatments. This Birdcage device emulates a small-scale MRI machine, producing the same 64 MHz frequency at much lower operating input power. In this work, we utilized a high-frequency simulation system (HFSS/EMPro) software to produce the birdcage structure for the required EMF parameters. The 64 MHz radiating frequency produced the scattering S11 parameter of −15 dbs. We obtained a SAR of 0.6 W/kg when an input power of 100 W was applied. The coil dimensions were found to be near 15 cm in height and 22 cm in diameter, which fits in wearable systems. We found that the distribution of the electric field and SAR radiate homogeneously over the simulated human head with good penetration into the brain, which proves to be an appropriate potential therapeutic strategy for Alzheimer’s disease.

## 1. INTRODUCTION

Alzheimer’s disease (AD) is a complex progressive brain disorder, which, unfortunately, to date, no effective drugs have been developed for treatment. It is the sixth leading cause of death in the United States and the fifth leading cause among those over age 65 [1]. AD causes the loss of memory and mental deterioration. It is also the most common cause of dementia associated with a progressive neurodegenerative disorder, with a prevalence of 44 million people throughout the world in 2015, and this figure is estimated to double by 2050 [2]. AD is a disease that is attributed to the accumulation of amyloid-*β* (A*β*) peptide, a decrease in acetylcholine levels, and a reduction of cerebral blood flow [3].

There have been numerous efforts with pharmacological treatments [3] such as acetylcholinesterase inhibitors and N-methyl-D-aspartate (NMDA) receptor antagonists, and etiology-based treatments such as secretase inhibitors, amyloid binders, and tau therapies. Despite extensive research in the treatment and management of AD, it is highly unlikely that any one drug will successfully treat this disease [4].

Over the past several decades, researchers have investigated the use of radiation as an alternative treatment. While bio-electromagnetic medicine presents the most important diagnostic tools and therapeutic modalities in modern medicine. There are still major issues regarding their effects on the central nervous system (CNS). Recently, research suggested that low-dose non-ionizing electromagnetic field stimulation could be a valuable therapeutic tool for the treatment of neurodegenerative diseases [5, 6]. The advantages of electromagnetic fields over pharmacological treatments include 1) it easily crosses the blood-brain barrier, 2) it has intra-neuronal effects, 3) it has homogeneous field distribution on the whole brain, 4) it is not dependent on blood circulation to reach neurons, 5) and it has high bioavailability compared to pharmacologic agents [7].

With no curative medications available in the market, recent efforts from the joint medical and engineering team at Indiana University Purdue University Indianapolis (IUPUI) have been pursued. We have investigated the use of non-ionizing, non-invasive, low power, low-frequency EMF for the treatment of AD patients via radio-frequency devices in efforts to decrease the toxic A*β* levels, which is considered to be the cause of AD. In this study, we utilized HFSS to produce the antenna parameters and the E-field distribution following the preliminary results reported in our recent paper [8].

The idea of the RF birdcage coil used here is based on the medical diagnostic MRI system for high-quality anatomical images with highly uniform magnetic and electric fields [9, 10]. The coil also features a high signal-to-noise ratio, design flexibility, and the ability to be designed for multi-resonance operation. The birdcage coil may be modeled as a closed ladder network composed of identical cascaded segments of inductive and capacitive elements [11]. The multiple closed current loops in the birdcage coil may give equivalencies to low pass, band pass, and high pass filter structures. The multiple cascaded identical segments will result in several resonance frequencies, also known as resonance modes. This depends on the number of legs N, the inductance of each leg and end ring segments, and the lumped capacitance of both the legs and the end ring [11]. The existing solutions involve complex mathematical formulation, also limited to the determination of the resonance characteristics of the birdcage coil. In practice, this may require impedance matching circuits with the birdcage RF coil, following the mathematical model done in a recent paper [12].

## 2. THE DEVICE MODEL

**Figure 1.**
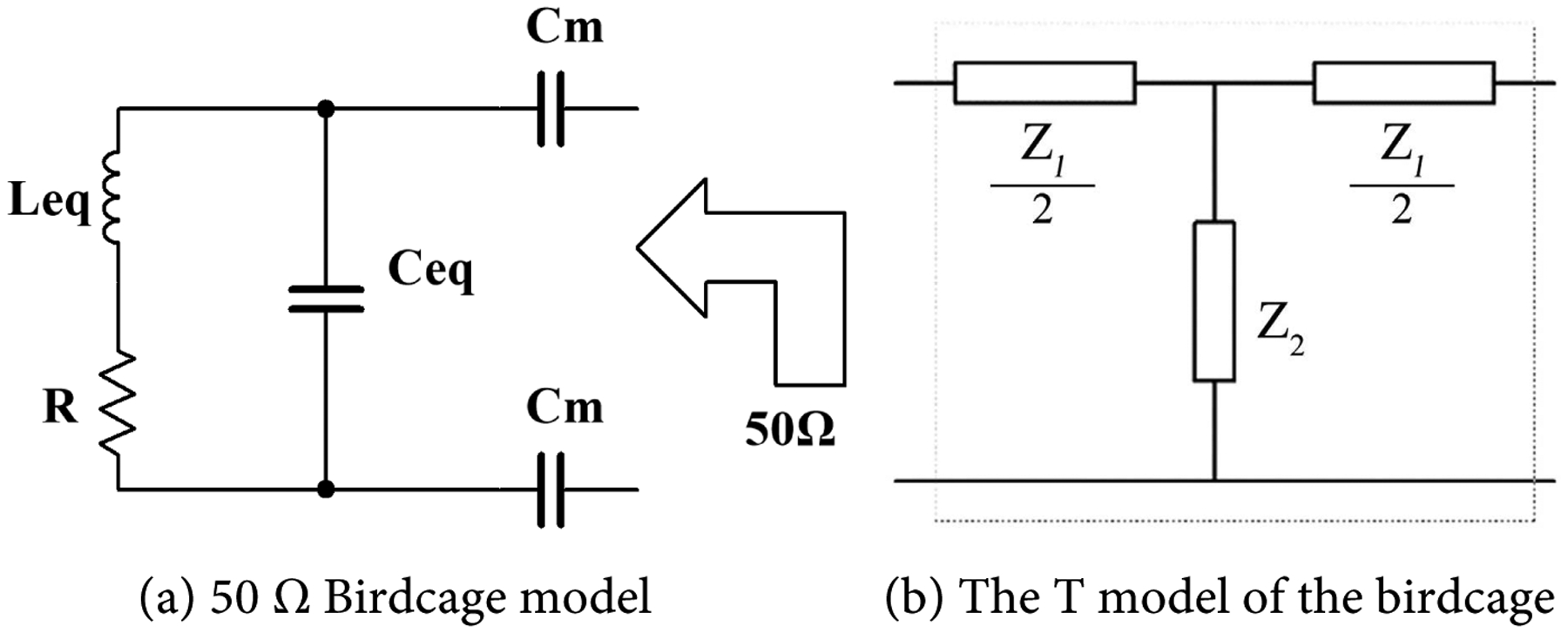
The birdcage model.

The simulated birdcage device was designed to provide a SAR of 0.6 W/Kg, and a frequency of 64 MHz for a proper time constant that produces a small change in temperature within a fraction of m°K. With the birdcage model given in Figure 1, and the radiating frequency, *f*_0_ will be given by:

(1) $$f_{0}=\frac{1}{2\pi \sqrt{L_{eq}C_{eq}}}$$

If *L*_*eq*_ is chosen to be 173.7 nH, and *C*_*eq*_ to be 35.6 pF, the device will radiate at *f*_0_ = 64 MHz.

The controlling equations for these capacitances and inductances are given as:

(2) $$C_{eq}=\frac{2\alpha \beta^{2}C_{t}}{N}$$

Choosing *C*_*t*_ =142.4 pF, *C*_*eq*_ = 35.6 pF.

The values of *L*_*rings*_ and *L*_*strip*_ may be estimated from the equations:

(3) $$L_{\text{end}\;\text{rings}}=\frac{\mu_{0}l}{2\pi }\left(\text{ln}\left(\frac{2l}{a}\right)-1\right)$$

(4) $$L_{\text{strip}}=\frac{\mu_{0}l}{2\pi }\left(\text{ln}\left(\frac{2l}{w}\right)+\frac{1}{2}\right)$$

For the model shown in Figure 1,

(5) $$L_{eq}=\frac{Z_{in}}{j\omega }$$

(6) $$Z_{in}=\frac{Z_{c}\text{sinh}\left(\frac{N\lambda }{2}\right)}{2\text{sinh}\left(\frac{\lambda }{2}\right)\text{sinh}\left[(N-1)\left(\frac{\lambda }{2}\right)\right]}$$

(7) $$Z_{c}={\left(\frac{Z_{1}}{2}\right)}^{2}+Z_{1}Z_{2}$$

(8) $$\lambda ={\text{cosh}}^{-1}\left(1+\frac{Z_{1}}{2Z_{2}}\right)$$

Given number of rings *N* = 16, the spatial phase factor *ϕ*, can be determined from:

(9) $$\phi =\frac{\pi }{N}$$

The spatial phase factor *ϕ*, in this case will equal 196.35 m rad.

The value of *a* that may be used in Equation (3) above can be determined from:

(10) $$\alpha =\{\begin{array}{ll} 1, & \text{for}\;\text{low}\;\text{pass}\\ 2{\text{sin}}^{2}\phi , & \text{for}\;\text{high}\;\text{pass} \end{array}$$

With high pass filter consideration, *α* = 2sin^2^ *ϕ*, where *ϕ* is given in Equation (9) above.

The value of *β* as given in Equation (2) above can be determined from

(11) $$\beta =\frac{1}{2}\sum_{n=1}^{N}\left|\text{cos}\left[\frac{\pi }{n}(2n+1)\right]\right|$$

With *N* = 16, the value of *β* will = 5.126. Table 1 gives relation between *N* and *β*.

The above equations will lead to values:

$$C_{t}=142.4\;\text{pF}$$

$$C_{eq}=35.6\;\text{pF}$$

$$L_{\text{end}\;\text{rings}}=30\;\text{nH}$$

$$L_{\text{strip}}=208\;\text{nH}$$

Considering the resistance of the cage coil, *R*_*coil*_ is given by

(12) $$R_{\text{coil}}=2\pi BL$$

where *B* is the 3 db frequenct. Considering 3d bs = 25 KHz. The quality factor of the coil may be estimated from the following equations:

(13) $$Q_{\text{empty}}=\frac{f_{c}}{f_{2}-f_{1}}=\frac{2\pi f_{0}L}{R_{\text{coil}}}$$

(14) $$Q=\frac{Q_{\text{empty}}}{Q_{\text{load}}}=\frac{R_{\text{coil}}+R_{\text{samp}}}{R_{\text{coil}}}$$

(15) $$\frac{1}{2}C_{m}=\sqrt{\frac{C_{eq}}{2\pi f_{0}Q_{\text{load}}50}}$$

Following the above equations, *C*_*m*_ is estimated to be 20.37 pF.

The change in temperature when the system reaches the 0.6 *SAR* value can be estimated from:

(16) $$\Delta T_{R}=\frac{c_{p}\Delta T}{\Delta SAR}$$

where Δ*T*_*R*_ is the rise time, Δ*T* is the change in temperature based on the value of Δ*SAR* (which is 0.6 SAR in this case), and *c*_*p*_ is the specific heat capacity.

The rise time, Δ*T*_*R*_ is given by:

(17) $$\Delta T_{R}=\sqrt{2\pi }\tau$$

Where *τ* is the time constant given by:

(18) $$\tau =\frac{L}{R}=RC$$

The specific heat capacity in this case is taken *c*_*p*_ = 3.46 kJ.

The above equations estimate the time constant and temperature values equal:

$$\Delta T=0.25\;\text{mK}\;\text{and}\;\Delta T_{R}=1.44\;\text{s}$$

## 3. SIMULATION RESULTS AND DISCUSSIONS

**Figure 2.**
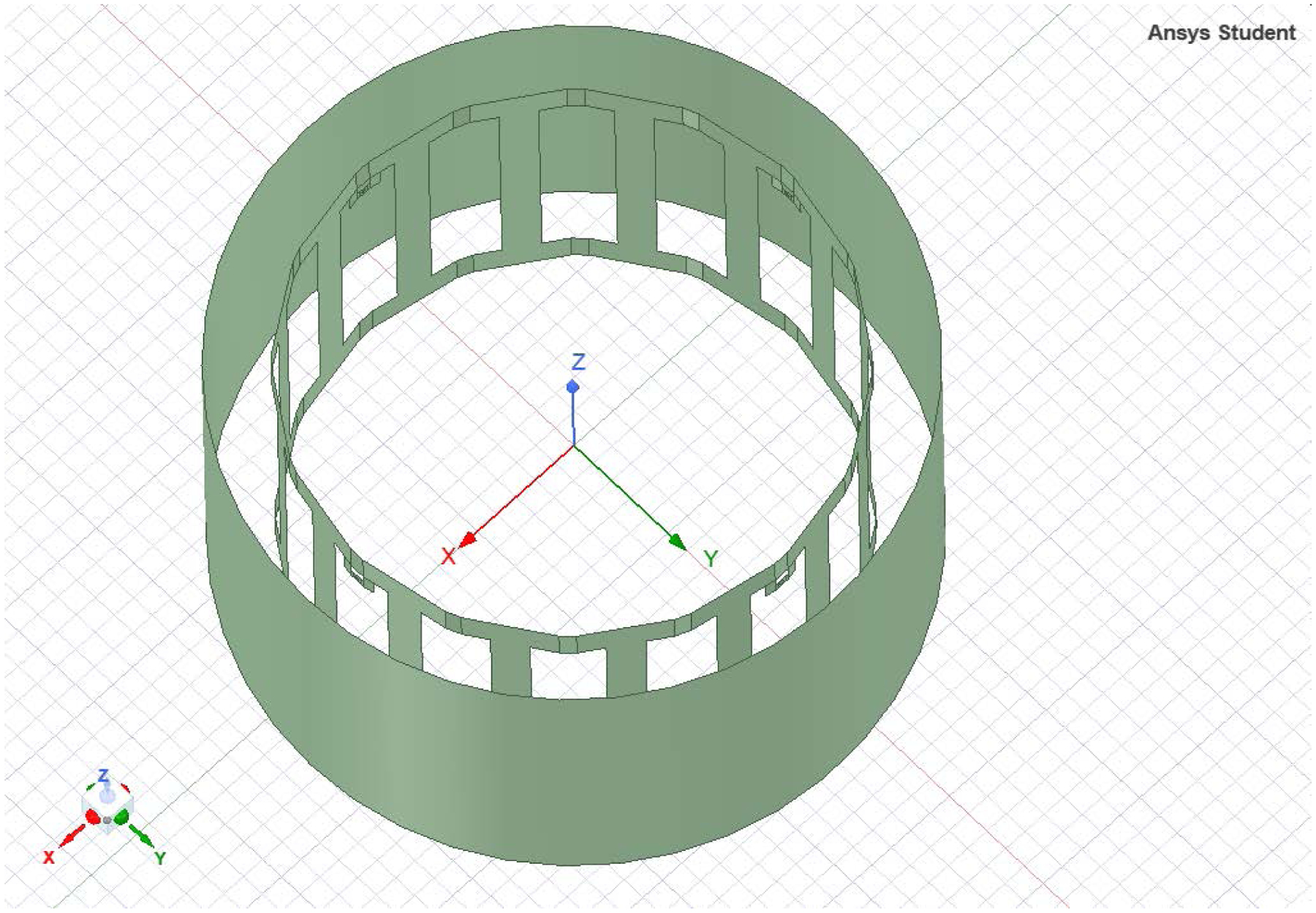
The birdcage structure.

**Figure 3.**
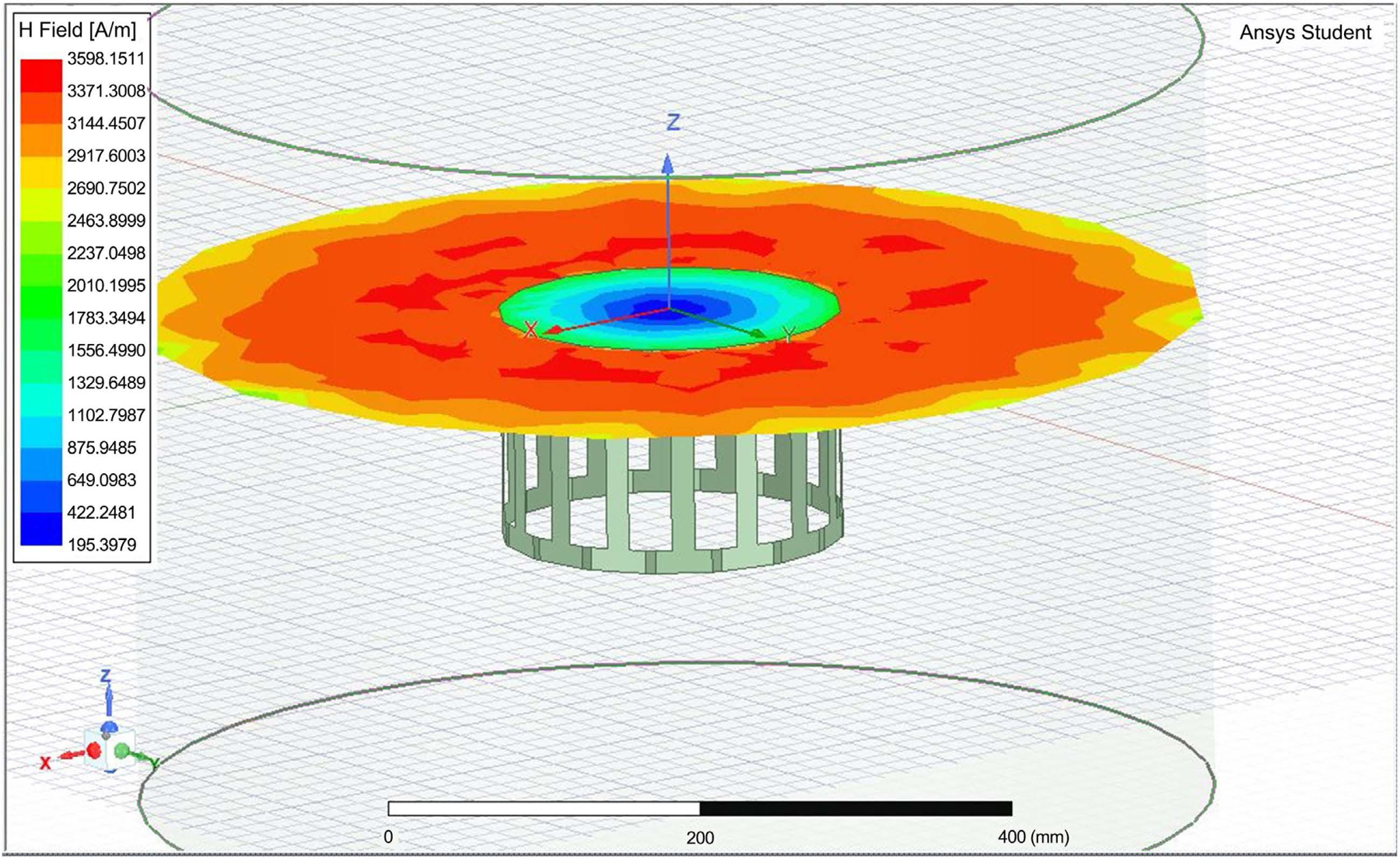
Displaced conductors more than 10 mm will cause H-field and accordingly EM energy to escape from the Birdcage system.

HFSS software was used in simulating the birdcage coil antenna to estimate the electric field and SAR distribution inside a simulated human head and brain. We estimated a birdcage with a diameter of 22 cm and a height of 15 cm to achieve the required SAR of 0.6 W/kg and a relative homogeneous E-field distribution inside the simulated coil. The birdcage was designed with a distance of 16 and 5 mm between wires. Figure 2 shows the coil structure. Figure 3 shows the H-field with wider shield with the E-field distribution. The distance between wires was critical in controlling the E-field distribution. Further distance may cause escape of the field outside the coil. We use Matlab for the mathematical model described above, with the ratio between the birdcage coil radius to shield of 4:5, the shield radius was equal to 1.25× the birdcage radius. The shield radius, Sr was near 6-inch diameter.

**Figure 4.**
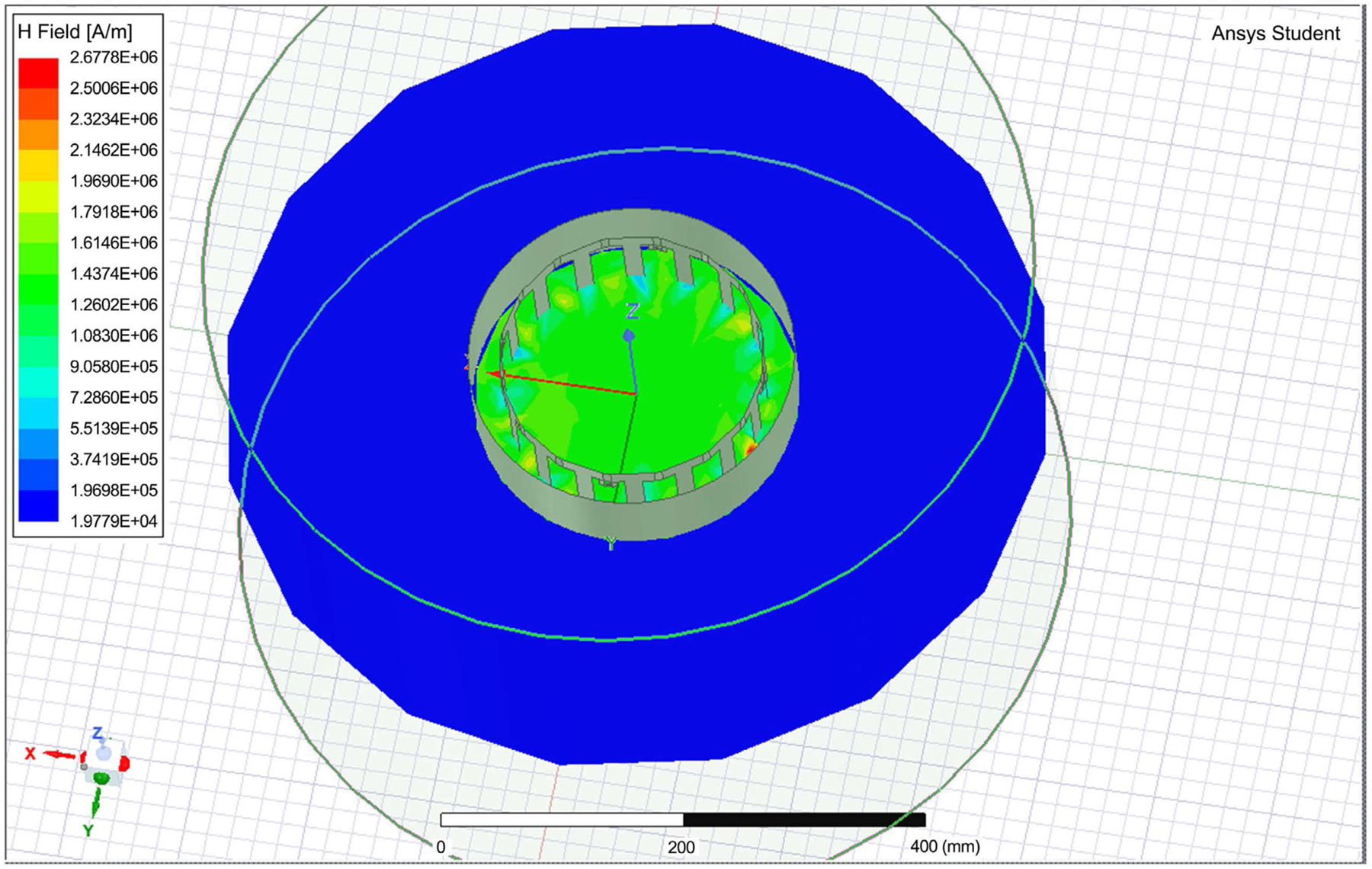
The H-field distribution inside the Birdcage coil.

**Figure 5.**
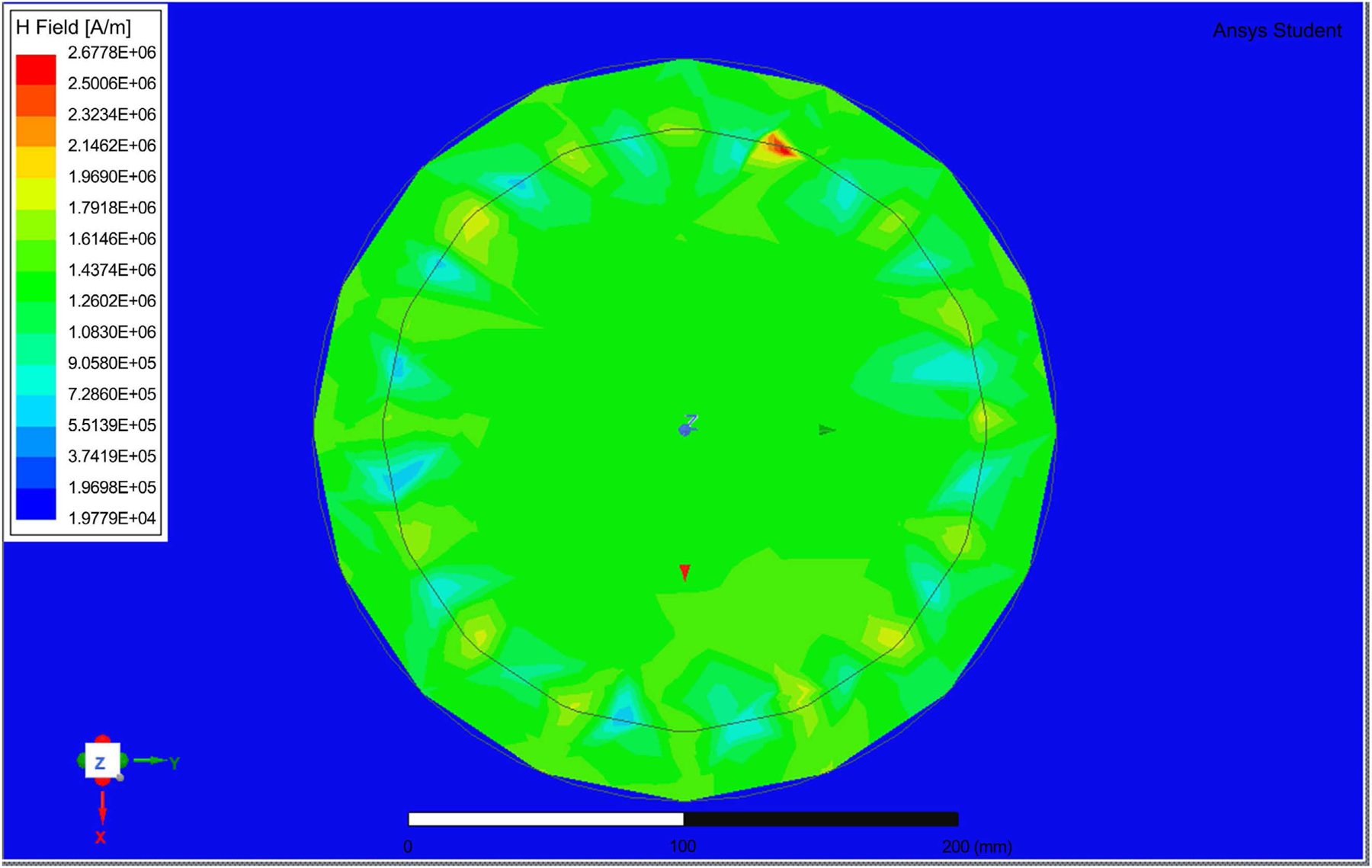
The Field distribution inside showing the shield coil.

**Figure 6.**
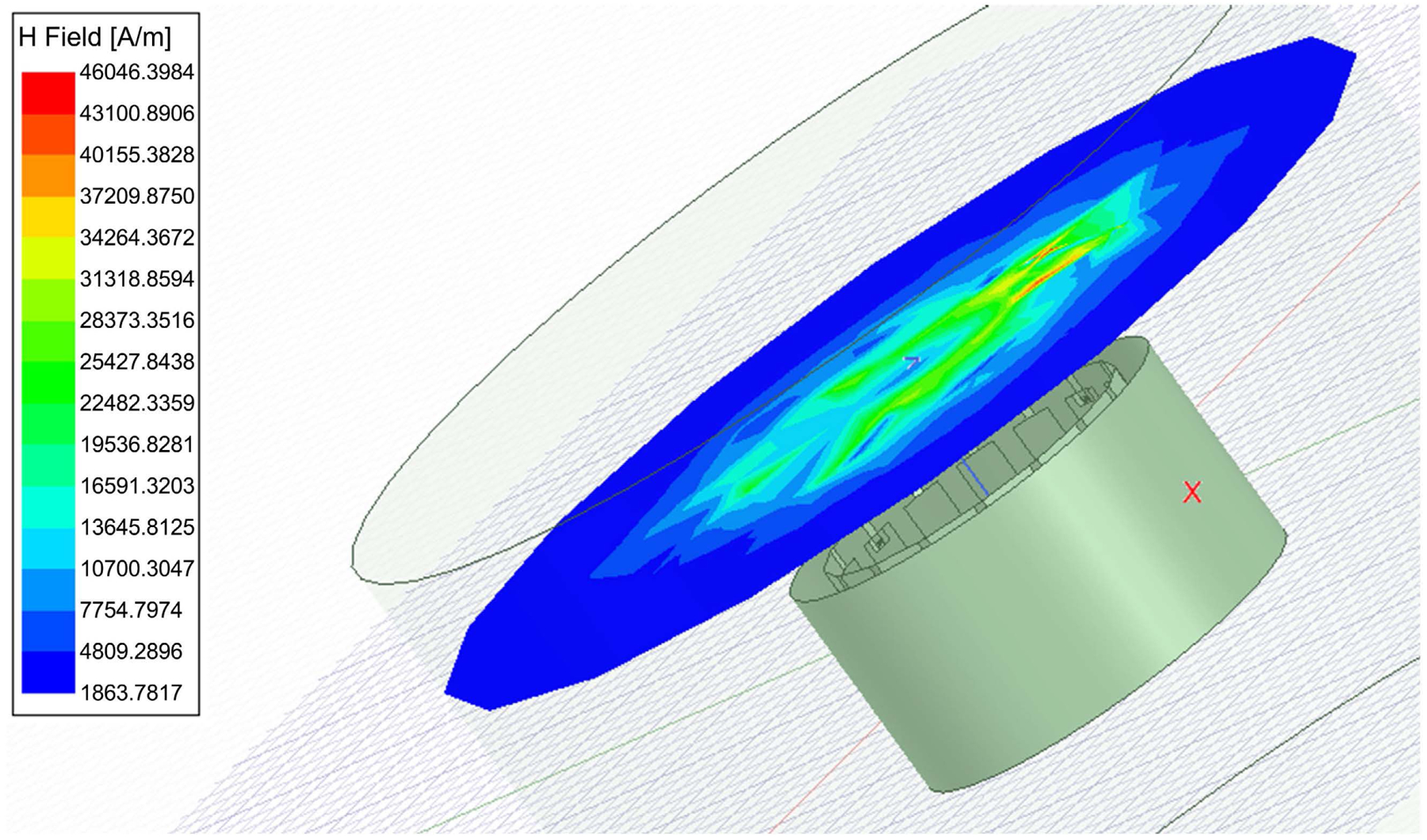
A different setting of a Birdcage coil simulation that may be accommodated for wearable devices. Notice H-field is a little above the birdcage coil.

As it can be seen the value of H = 1.2 × 10^6^, occurring almost uniformly inside the coil. This matches with the Matlab simulation, with H-field needed to be 1.19 M Amps/Meter, with energy consumption reaches near 100 W in a short time. Figure 4 shows the field distribution within the tissue inside the coil. Figure 5 is a zoom in view that shows the shield coil of the birdcage. Figure 6 shows the H-field distribution with different patient setting that may be appropriate for wearable system.

**Figure 7.**
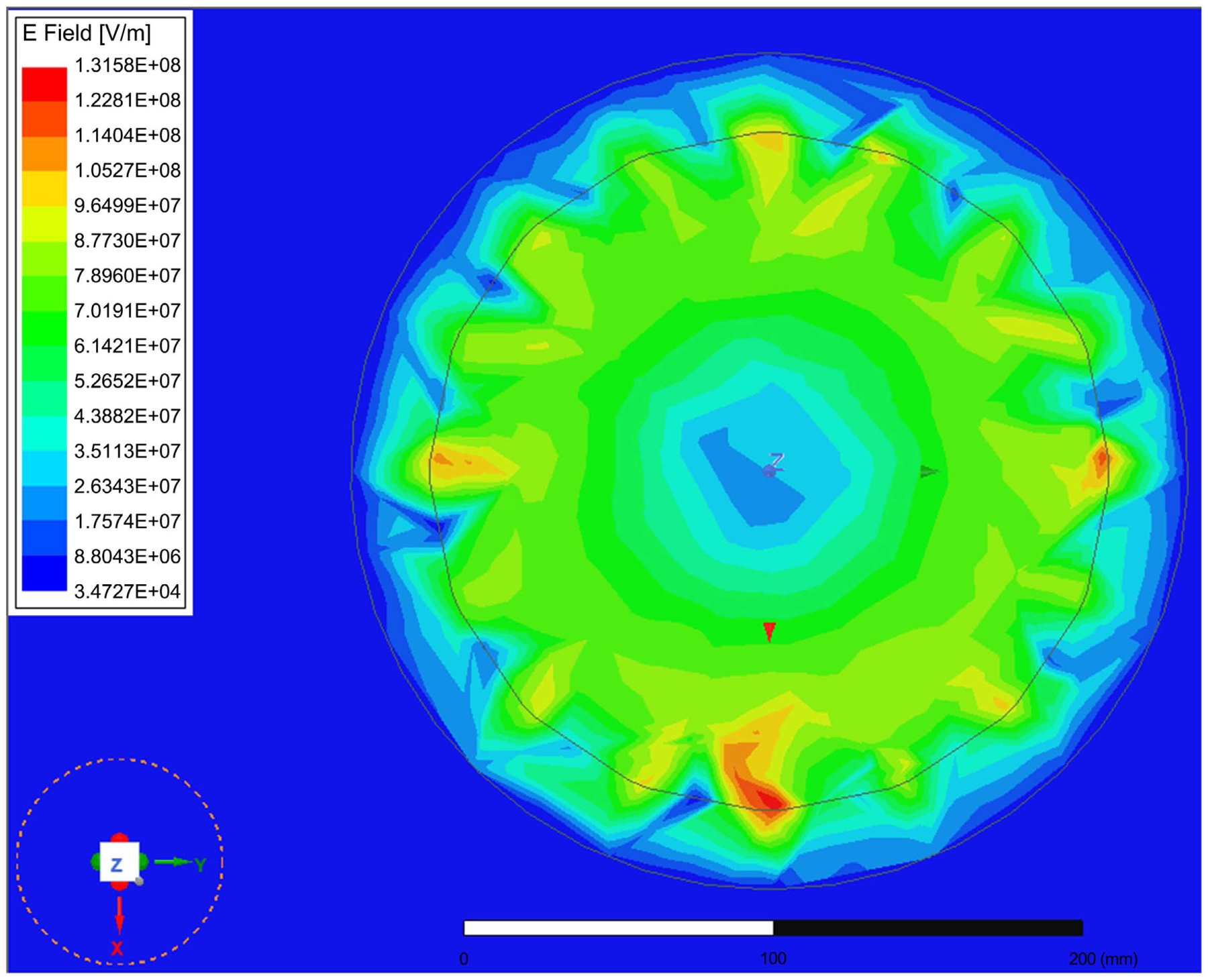
The E-Field distribution over the head phantom.

The E-Field distribution is given in Figure 7. The field is uniform inside the simulated birdcage coil. The simulation results showed that the magnetic energy impacting the SAR values is focused inside of the coil with H values near 1.25 × 10^6^ A/m. This makes B = 0.58 T. The data shows that the magnetic field quickly attenuates outside the coil. The value of H above the coil was near 22 KA/m, making B near 27 mT. The high localized fields near the end rings are of narrow regions, and not propagating to the inside. These are resulting from boundary values of the fields near the rings.

## 4. CONCLUSION AND FUTURE WORK

In this work, a Birdcage coil antenna was proposed to produce the required EMF parameters of a SAR of 0.6 W/kg, and a frequency of 64 MHz following the preliminary biological data presented in our previous study [5]. The HFSS simulation results showed that the Birdcage device dimensions may be adopted as a wearable device for the AD treatment. The simulation showed uniform distribution for the E and H fields in the middle of the coil, suggesting uniform power density applied to a simulated human head inside the coil. The simulation with the head phantom and the practical model of the system including hair factor are reserved for future considerations.

## Figures and Tables

**Table 1.** *β* Values as a function of the birdcage legs number *N*.

***N***	4	8	12	16
*β*	1.414	2.613	3.863	5.126
